# Flavonoid GL-V9 suppresses invasion and migration of human colorectal cancer cells by inhibiting PI3K/Akt and MMP-2/9 signaling

**DOI:** 10.7150/jca.58710

**Published:** 2021-06-01

**Authors:** Ye Gu, Jiejie Yu, Cong Ding, Yifeng Zhou, Jiangfeng Yang, WeiPing Yu, Xiaofeng Zhang, Haitao Huang

**Affiliations:** 1 Department of Gastroenterology, Key Laboratory of Clinical Cancer Pharmacology and Toxicology Research of Zhejiang Province, Affiliated Hangzhou First People's Hospital, Zhejiang University School of Medicine, Hangzhou, Zhejiang 310006, P.R.China.; 2 Department of Gastroenterology, The Fourth Clinical Medicine College, Zhejiang Chinese Medical University, Hangzhou, Zhejiang 310006, P.R.China.; 3 Hangzhou Hospital & Institute of Digestive Diseases, Hangzhou, Zhejiang 310006, P.R.China.; 4 Key Laboratory of Integrated Traditional Chinese and Western Medicine for Biliary and Pancreatic Diseases of Zhejiang Province, Hangzhou, Zhejiang 310006, P.R.China.; 5 Department of Pathophysiology, Medical school of Southeast University, Nanjing, Jiangsu 210009, P.R. China.

**Keywords:** GL-V9, colorectal cancer, invasion, matrix metalloproteinases, PI3K/Akt

## Abstract

Tumor distant metastasis is the primary cause of death in colorectal cancer (CRC) patients. GL-V9 is a newly synthesized flavonoid derivative with several beneficial biological functions including anti-tumor and anti-inflammation. However, the anti-metastatic effect of GL-V9 and related mechanisms in CRC remains unknown. In this study, the anti-invasive and anti-migratory activities of GL-V9 were investigated in CRC cells. Using MTT assay, cell wound healing assay, and transwell migration assay, we showed that GL-V9 suppressed CRC cell viability, migration, and invasion in a concentration-dependent manner. In addition, the protein expression levels as well as activities of matrix metalloproteinase-2 (MMP-2) and matrix metalloproteinase-9 (MMP-9) were significantly reduced after GL-V9 treatment. Further analysis of the underlying mechanism revealed that GL-V9 inhibited PI3K/Akt signaling pathway upstream of MMP-2 and MMP-9. In conclusion, our study demonstrated that GL-V9 could suppress CRC cell invasion and migration through PI3K/Ak and MMP-2/9 axis. Therefore, GL-V9 might be a potential novel therapeutic agent against CRC metastasis.

## Introduction

Colorectal cancer (CRC) is a growing threat to human health which ranks the third commonly diagnosed malignancy and the second leading cause of cancer death worldwide 1,2. Tumor relapse and distant metastasis following conservative surgery are among the major causes of mortality in CRC patients and signify a poor prognosis 3. Therefore, it is urgent to develop novel anti-metastasis therapeutic agents in order to improve the overall survival of CRC patients.

Cancer invasion and metastasis involve multistep processes including genetic changes and phenotypic alterations of cancer cells 4. Among these processes the degradation of environmental barriers, such as the extracellular matrix (ECM) and basement membrane is a key event contributing to cancer cell malignancy 5. Belonging to the family of zinc-dependent endopeptidases, matrix metalloproteinases (MMPs) can collectively degrade nearly all extracellular matrix components and destroy the histological barrier of cancer cell invasion, enabling cancer cells to escape from primary tumor and migrate through basement membrane of blood vessels and connective tissues 6,7. Among all the MMP proteins identified so far, MMP-2 and MMP-9 are considered to be the most crucial members involved in tumor migration and invasion 8. Overexpression of MMP-2 and MMP-9 are frequently detected in CRC and predict poor prognosis of CRC patients 9-11. The expression levels of these two proteins are highly correlated with tumor metastasis potential 9-11. The MAPK/ERK and PI3K/Akt signaling pathways are prominent upstream regulators of MMP-2 and MMP-9. Accumulated evidences have shown that activation of MAPK/ERK and PI3K/Akt promotes CRC invasion and metastasis via up-regulating MMP-2 or MMP-9 expression 12-15. Therefore, the MAPK/ERK/MMPs or PI3K/Akt/MMPs cascades may serve as promising targets for anti-metastasis drugs 12.

GL-V9 (5-hydroxy-8-methoxy-2-phenyl-7-(4-(pyrrolidin-1-yl) butoxy) 4H- chromen-4-one) is a flavonoid derivative synthesized from the natural product wogonin 16. The pro-apoptotic, anti-inflammatory, anti-invasive and anti-metastatic effects of GL-V9 have been reported in breast cancer, gastric cancer, and hepatocellular carcinoma 16-19. However, whether GL-V9 can affect CRC metastasis has remained unclear. In this study, we examined the *in vitro* effects of GL-V9 on CRC cell viability, invasion and migration. The relevant pathways and underlying molecular mechanisms were further investigated. Our study may provide new evidences for the potential capability of GL-V9 in clinical treatment of CRC.

## Materials and methods

### Reagents

GL-V9 (C_24_H_27_NO_5_, MW: 409.47, purity > 99%) was kindly provided by Dr. Bin Di (China Pharmaceutical University, China) and dissolved in dimethyl sulfoxide (DMSO, Sigma-Aldrich, St. Louis, USA) to 0.1 M as a stock sulution and stored at -20°C. For cellular experiment, GL-V9 was dissolved in sterilized dimethylsulfoxide (DMSO) and diluted with cell culture medium to different final concentrations. The final DMSO concentration did not exceed 0.1% v/v (volume-to-volume ratio) throughout the study. The PI3K/Akt signaling activator Insulin-like growth factor-1 (IGF-1) and the specific PI3K inhibitor LY294002 was obtained from Beyotime Biotechnology Corporation, Shanghai (Shanghai, China).

Primary antibodies against PI3 Kinase p85, Akt, p-Akt (Ser473), p38 MAPK, p-p38 MAPK (Thr180/Tyr182), ERK1/2, p-ERK1/2 (Thr202/Tyr204), MMP-2, MMP-9, and β-actin were obtained from Cell Signaling (Danvers, MA, USA) and were used at a 1:1000 dilution. HRP-conjugated secondary antibodies were obtained from Cell Signaling (Danvers, MA, USA) and were used at a 1:2000 dilution. MTT (3-(4,5)-dimethylthiahiazo(-z-y1)-3,5-diphenytetrazoliumromide) was obtained from Sigma-Aldrich (St. Louis, MO, USA).

### Cell culture

Four human CRC cell lines (HCT116, SW480, SW620 and LS174T) and one normal human colon epithelial cell line FHC were purchased from the American Type Culture Collection (Manassas, VA, USA). Cells were cultivated in DMEM medium (Gibco, Carlsbad, CA, USA) supplemented with 10% heat-inactivated fetal bovine serum (FBS, Gibco, Carlsbad, CA, USA), 100 U/ml penicillin, and 100 mg/l streptomycin. All cells were grown in a stable environment with 5% CO_2_ at 37 °C.

### Cell viability assay

The effect of GL-V9 on the viability of CRC cells as well as normal colon epithelial cells was determined by MTT assay. Briefly, cells were seeded at a density of 5 × 10^3^ cells/well onto 96-well microtiter plates containing 100 μl of culture medium and exposed to GL-V9 at different concentrations or equivalent amounts of DMSO as negative control. After treatment for 12, 24 or 48 h, 20 μl of MTT solution (5 mg/ml) was added to each well. The plate was incubated at 37 °C for 4 h in a humidified atmosphere with 5% CO_2_. Then supernatants were removed carefully and 100 μl DMSO was added to each well. The absorbance was measured at 570 nm using a microplate autoreader (Bio-Rad, Hercules, CA, USA). The survival rate (%) was calculated using the formula: Survival Rate (%) = OD_treated_/OD_control_ × 100%. The inhibition ratio (%) was calculated using the formula: Inhibition Ratio (%) = [(OD_control_-OD_treated_)/OD_control_ × 100%. The IC50 value, which is the concentration that caused 50% inhibition of cell viability, was calculated using GraphPad Prism v6.0 (GraphPad software, Inc. California, US). The OD values are the average absorbance of three parallel experiments.

### Wound healing assay

Cells were seeded at a density of 1 × 10^6^ cells/well onto six-well plates and cultured in medium supplemented with 10% FBS (fetal bovine serum). After the cells grown to 80 - 90% confluence, wounds were created in lines across each well using a 10 μl sterile pipette tip. The plates were then washed three times with PBS to remove cell debris and replaced with 2 ml of serum-free fresh medium with or without different concentrations of GL-V9. Cells were allowed to grown for 24 h, during which time wound margins were photographed and migration was monitored using an inverted microscope. Images were captured using an image-analyzing frame-grabber (LG-3 Scientific Frame Grabber; Scion, Frederick, MD) and analyzed with image analysis software (NIH Image 1.55). The unfilled scratched zones were quantified by measuring the distance between the advancing margins of cells in five randomly selected microscopic fields (×200) at each time point. The experiment was performed in triplicates.

### Cell invasion assay

Cell invasion assays were conducted using the transwell assay (Corning Costar, Cambridge, MA, USA). Matrigel was diluted to 5 mg/ml with DMEM serum-free medium and applied to 8-μm-pore polycarbonate membrane filters of the chamber. Cells treated with or without different concentrations of GL-V9 for 24 h were seeded onto the upper chamber at a density of 5 × 10^4^ cells/well. Then, 300 μl of medium with 10% FBS (fetal bovine serum) was added into the lower chamber. After incubation for 24 h at 37 °C in5% CO_2_, cells in the lower chamber were fixed with 1% paraformaldehyde and stained with haematoxylin after removal of non-invasive cells inside the chamber with cotton swabs. The number of cells migrated through the chamber was counted in five randomly selected fields (×200) under a microscope. The experiment was performed in triplicates.

### Cell attachment assay

Cell adhesion assays were performed in 96-well culture plates. The plates were coated with 5 mg/ml of fibronectin (Sigma, St. Louis, MO, USA) overnight at 4 °C and blocked with 1% bovine serum albumin (BSA) in PBS at 37 °C for 4 h. Cells pre-treated with different concentrations of GL-V9 or without GL-V9 treatment were re-suspended in serum-free DMEM medium at a density of 5 × 10^5^ cells/ml. Then 100 μl of cell suspension was seeded into each well. After incubation at 37 °C for 1 h, unattached cells were removed by 3 × PBS washes and the adherent cells were quantified by the MTT assay. The experiment was performed in triplicates.

### Gelatin zymorgraphy

The activities of MMP-2 and MMP-9 were detected by gelatin zymography assay as previously described 20. Cells were exposed to GL-V9 at different concentrations for 24 h, and the supernatant was collected and mixed with loading buffer at a ratio of 3:1 without heating or reduction. The loading volume of each conditioned medium sample was normalized according to the concentration of enzyme detected by BCA assay. The prepared samples were then loaded onto 10% SDS-PAGE with 0.1% gelatin before electrophoresis. The gel was washed in 50 mM Tris-HCl buffer (pH 7.6) containing 2.5% (v/v) Triton X-100 for 30 min on a shaker to remove SDS and incubated for 24 h at 37 °C in developing buffer (50 mM Tris-HCl, pH 7.6, 5 mM CaCl_2_, and 1 mM ZnCl_2_). Finally, the gel was stained with 0.1% Coomassie Brilliant Blue G250 for 1 h and de-stained in 10% acetic acid and 10% methanol.

### Western blot

Cells were lysed on ice in Radio immunoprecipitation assay (RIPA) buffer. Protein concentrations were determined using the Bradford reagent (BioRad, Hercules, CA, USA). Cellular lysates containing equal amounts of total protein were resolved on 12% SDS polyacrylamide gel and electrotransferred to polyvinylidene fluoride membranes (ImmobilonP; Millipore, Bedford, MA, USA). The membranes were then blocked with 5% non-fat dry milk in Tris-buffered saline and immunoblotted using the respective primary antibodies overnight at 4 °C. Finally, the appropriate HRP-conjugated secondary antibodies were applied and signals were detected by enhanced chemiluminescence (Pierce, Rockford, IL, USA) following the manufacturer's instructions.

### Statistical analysis

Statistical analyses were performed using SPSS version 20 (SPSS Inc., Chicago, IL, USA). All results shown represent mean ± S.D. from replicate experiments performed in a parallel manner. Cell migratory rate, invasive ability, and protein expression levels were compared using unpaired student's *t*-test (two-tailed) between two groups and one-way analysis of variance (ANOVA) followed by Tukey's post-hoc test among three or more groups.

## Results

### GL-V9 inhibits the viability of CRC cells

**Figure 1:**
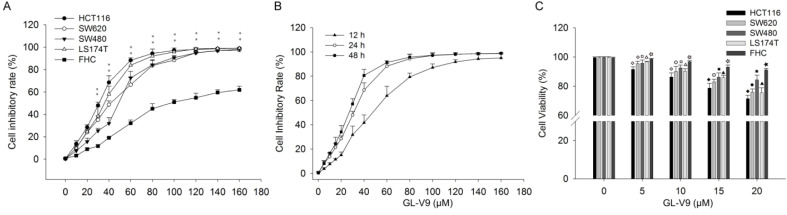
** Inhibitory effects of GL-V9 on the viabilities of CRC cells and normal human colon epithelial cells.** MTT assays were used to detect cell viability after GL-V9 treatment. (A) CRC cells HCT116, SW620, SW480, LS174T, and normal colon epithelial cells FHC were treated with 0-160 μM GL-V9 for 24 h. ** p<0.001 for all the examined cell lines. (B) CRC cells HCT116 were treated with 0-160 μM GL-V9 for 12, 24, and 48 h. (C) Survival rates of CRC cells HCT116, SW620, SW480, LS174T and normal colon epithelial cells FHC treated with 0, 5, 10, 15, and 20 μM GL-V9 for 24 h. GL-V9 showed marginal cytotoxic effect on FHC cells even at a concentration of 20 μM (inhibition rate < 10%). Each error bar represents the mean ± S.D. of three replicate samples. Symbols on each error bar represent the statistical significance of each cell line. HCT116: ◇,* p*<0.05, ◆, *p*<0.001. SW620: ○,* p*<0.05; ●, *p*<0.001. SW480: □, *p*<0.05; ■, *p*<0.001. LS174T: △, *p*<0.05; ▲, *p*<0.001. FHC: ☆,* p*<0.05; ★, *p*<0.001.

We first investigated the effect of GL-V9 on the viability of CRC cells and normal colon cells. GL-V9 treatment at various concentrations (0-160 μM) and durations (12, 24, 48 h) exhibited a dose- and time-dependent inhibitory effect on the viability and proliferation of different CRC cell lines, as determined by the MTT assay (Fig. 1). IC50 values of 24 h GL-V9-treatment for CRC cells HCT116, SW480, SW620, LS174T and normal cells FHC were 28.08 ± 1.36, 44.12 ± 1.54, 36.91 ± 2.42, 32.24 ± 1.60, and 81.89 ± 4.26 μM, respectively (Fig. 1A). Changes in cell viability under 24 h treatment of GL-V9 were similar to those under 48 h treatment, but were much greater than those under 12 h treatment (Fig. 1B). At concentrations lower than 20 μM, GL-V9 has marginal cytotoxic effect on normal colon cells. After 24 h treatment of GL-V9 at 20 μM, the inhibitory rates were (28.50 ± 2.25)%, (15.60 ± 3.15)%, (24.07 ± 2.14)%, and (24.50 ± 3.36)% for HCT116, SW480, SW620 and LS174T cells. However, the inhibitory rate for FHC cells was only (8.87 ± 1.21)% (Fig. 1C). Therefore, the concentration range from 0 to 20 μM with rare effects on cell viability of normal cells was chosen for the subsequent experiments.

### GL-V9 inhibits the migration, invasion, and adhesion of CRC cells

**Figure 2:**
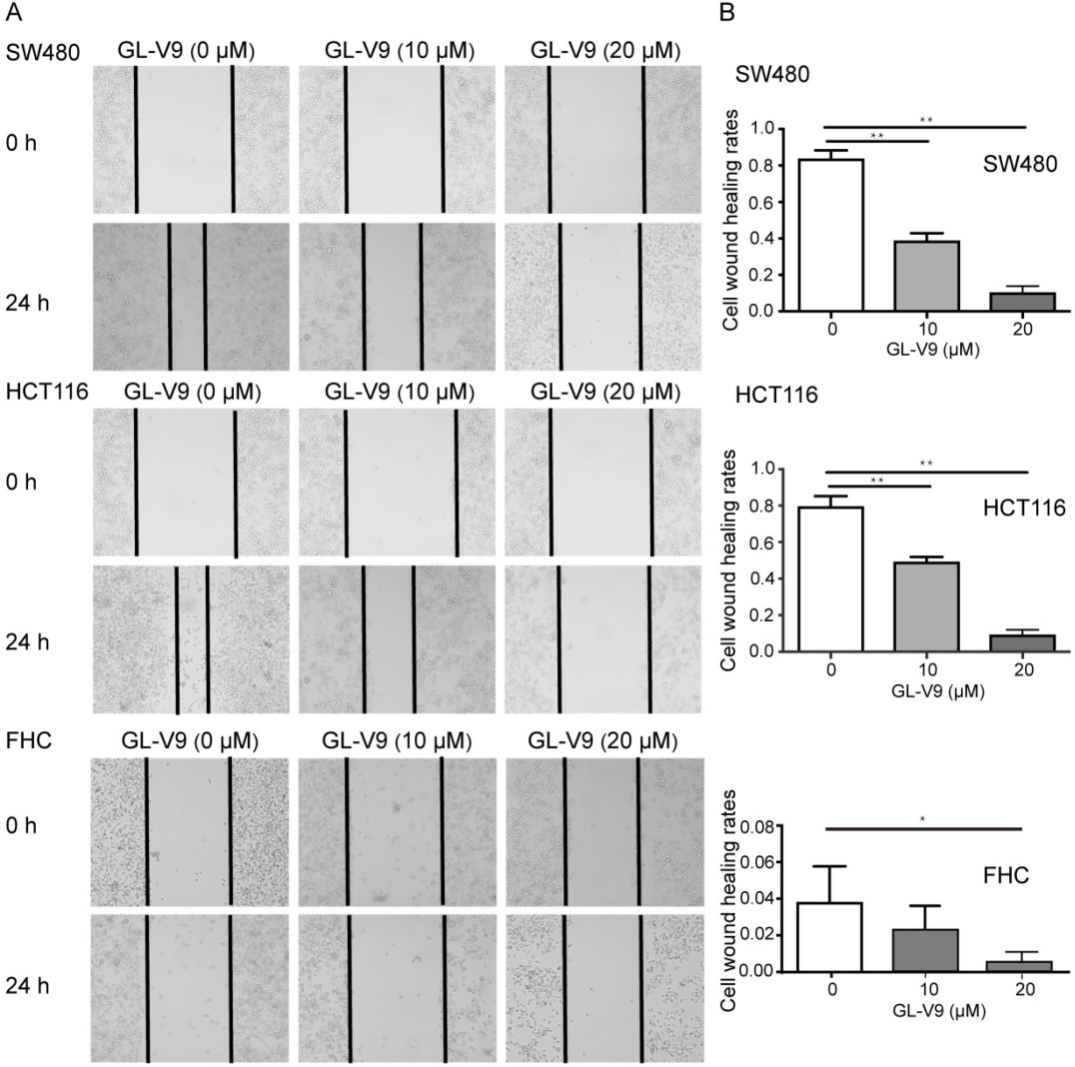
** Effect of GL-V9 on cell migration *in vitro*.** CRC cells HCT116 and SW480 treated with 10 μM and 20 μM GL-V9 for 24 h demonstrated significantly reduced migratory capacities, as indicated by wound healing assay. Normal colon epithelial cells FHC with much lower migration potential also showed reduced migration after GL-V9 treatment. The representative photographs (A) and quantification (B) are shown. Images were taken at 0 and 24 h. Each error bar represents the mean ± S.D. of three replicate samples. * *p*<0.05, ** *p*<0.001.

**Figure 3:**
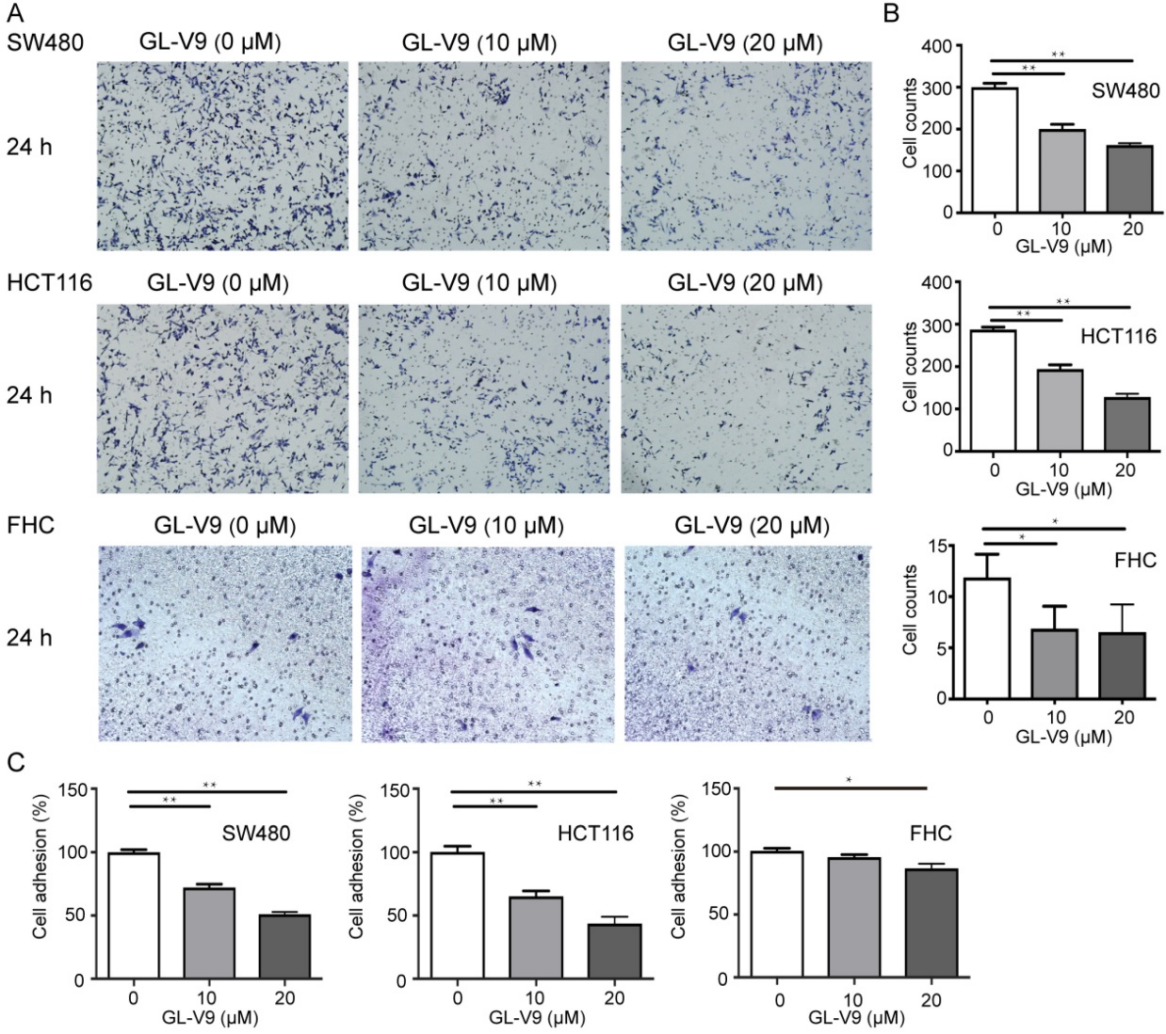
** Effect of GL-V9 on CRC cell invasion and adhesion *in vitro*.** (A-B) CRC cells HCT116 and SW480 treated with 10 μM and 20 μM GL-V9 for 24 h demonstrated significantly reduced migration and invasion through extracellular matrix as indicated by the transwell migration assay. Normal colon epithelial cells FHC with much lower invasive potential also showed reduced invasion through the extracellular matrix (ECM) after GL-V9 treatment, but with less reduction degree and statistical significance. The number of cells migrated through the ECM after 24 h was counted in five randomly selected (×200) microscopic fields. (C) GL-V9 treatment for 24 h at a concentration of 20 μM significantly reduced cell adhesion for both CRC cells HCT116 and SW480 and normal cells FHC, as indicated by cell attachment assay. Each error bar represents the mean ± S.D. of three replicate samples. * p<0.05; **, *p*<0.001.

CRC cell lines HCT116 and SW480 with the highest and lowest sensitivity to GL-V9 treatment were selected for cell migration, invasion, and adhesion assays. To compare the effects of GL-V9 on CRC cells and normal cells, the migration, invasion, and adhesion of normal colon cells FHC with or without GL-V9 treatment were also examined. Treatment of GL-V9 significantly reduced migratory abilities of CRC cells (*p* < 0.001; Fig. 2). After migration for 24 h, the extents of wound closure were (9.50 ± 2.59)% and (10.50 ± 3.30)% for HCT116 and SW480 cells treated with 20 μM of GL-V9, but were (79.67 ± 5.61)% and (83.83±4.49)% for HCT116 and SW480 cells without GL-V9 treatment (Fig. 2). In addition, transwell migration assay indicated that GL-V9 treatment led to remarkable decrease in the invasive capacities of CRC cells (*p* < 0.001; Fig. 3A, B). Compared with CRC cells, normal colon cells exhibited much lower migratory property. The extent of wound closure was only (0.038 ± 0.02)% for FHC cells without GL-V9 treatment and was (0.004 ± 0.01)% for FHC cells treated with 20 μM of GL-V9 (*p*=0.003; Fig. 2). Similarly, the invasive capacity of FHC cells was much lower than that of CRC cells. The number of FHC cells migrated through the extracellular matrix (ECM) was 11.83 ± 2.32 without GL-V9 treatment, but was only 6.5 ± 2.74 after treatment of 20 μM GL-V9 (*p*=0.005; Fig. 3A, B).

A key initial step of metastasis is the adhesion of cancer cells to extracellular matrix (ECM) components. Here we also examined the effect of GL-V9 on cell adhesion using the cell attachment assay. GL-V9 treatment significantly reduced CRC cell adhesion as compare with the control group (*p*<0.001). At a concentration of 20 μM, the inhibitory rates of cell adhesion were (56.63 ± 9.83)% for HCT116 cells and (48.97 ± 3.35)% for SW480 cells (Fig. 3C). For FHC cells, treatment of 20 μM GL-V9 also significantly reduced cell adhesion (*p=*0.005), but the inhibitory rates was only (14.02 ± 5.57)% (Fig. 3C). Collectively, our results demonstrated that GL-V9 significantly reduced CRC cell migration, invasion, and adhesion in a dose-dependent manner.

### GL-V9 suppresses the expression and activities of MMP-2 and MMP-9

**Figure 4:**
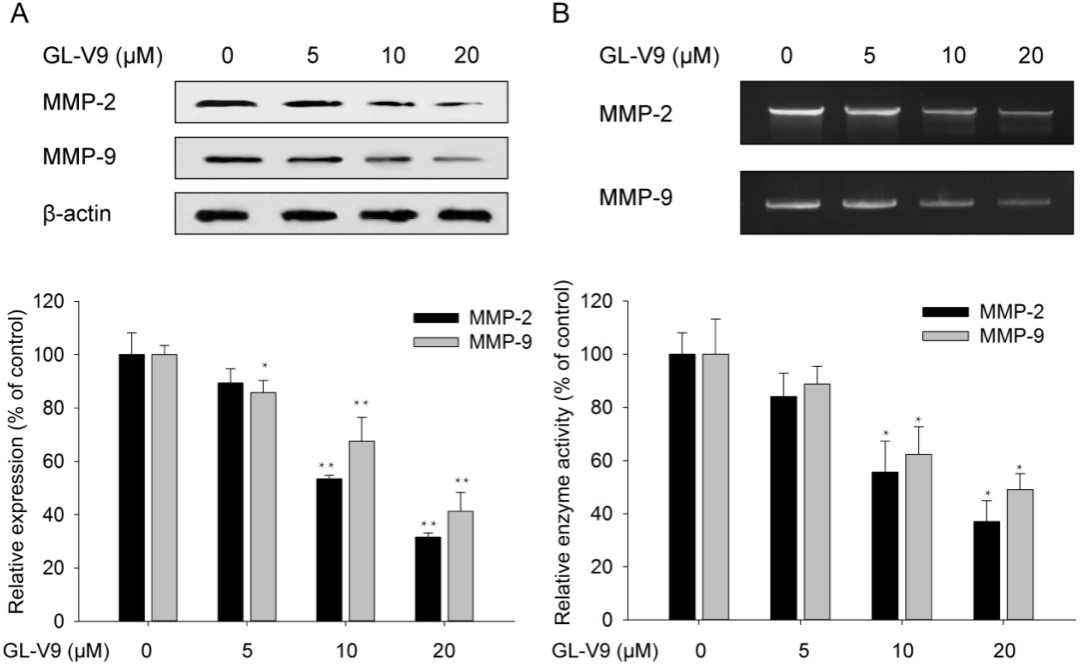
** Changes in the expression and activities of MMP-2 and MMP-9 after GL-V9 treatment.** (A) Treatment of 5, 10, and 20 μM GL-V9 for 24 h in HCT116 cells led to decreased MMP-2 and MMP-9 protein expression. Relative intensities of western blot bands were quantified using the ImageJ software. The expression levels were normalized by β-actin expression, which was used as internal control. (B) Treatment of 5, 10, and 20 μM GL-V9 for 24 h in HCT116 cells led to reduced activities of MMP-2 and MMP-9, as indicated by gelatin zymorgraphy. Each error bar represents the mean ± S.D. of three replicate samples. * *p* < 0.05, ** *p* < 0.01.

Matrix degrading proteinases play a pivotal role in the degradation of extracellular matrix (ECM), which further contributes to migration and invasion. Therefore, we examined the protein expression levels of MMP-2 and MMP-9 in HCT116 cells exposed to 5, 10, and 20 μM GL-V9 for 24 h. Results from western blot analyses showed that treatment of GL-V9 with a concentration ≥10 μM significantly reduced MMP-2 expression (*p*<0.001) and MMP-9 expression (*p*=0.0033) as compared with the control group (Fig. 4A). In addition, gelatin zymography analyses demonstrated that treatment of GL-V9 with a concentration ≥10 μM resulted in significant decreases in enzyme activities of MMP-2 (*p*=0.012) and MMP-9 (*p*=0.018) as compared with the control group (*p* < 0.05; Fig. 4B).

### GL-V9 suppresses PI3K/Akt signaling

**Figure 5:**
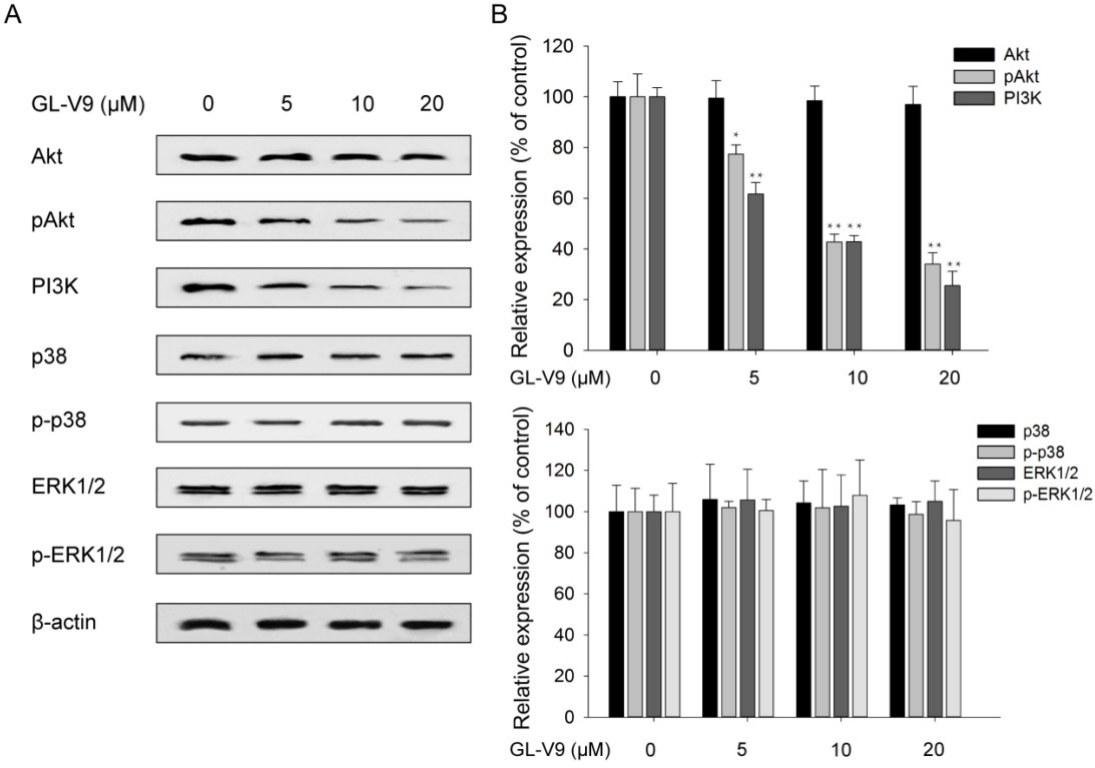
** Effects of GL-V9 on the PI3K/Akt and MAPK/ERK signaling pathways.** (A) Treatment of 5, 10, and 20 μM GL-V9 for 24 h in HCT116 cells inhibited PI3K/Akt activity in a dose-dependent manner, but did not affect the activity of MAPK/ERK signaling. PI3K expression and phosphorylated form of Akt were significantly reduced in GL-V9-treated cells, but expression of p38 MAPK and ERK1/2 in both the total and phosphorylated forms showed little change after GL-V9 treatment. (B) Quantification of the relative protein expression levels of PI3K, Akt, p-Akt, p38, p-p38, ERK1/2, and p-ERK1/2 in HCT116 cells treated with different concentrations of GL-V9, as compared with the control group. Each error bar represents the mean ± S.D. of three replicate samples. * *p* < 0.05, ** *p* < 0.01.

To explore the possible mechanism underlying the anti-cancer effect of GL-V9, we investigate the correlation between GL-V9 treatment and the activities of PI3K/Akt signaling and MAPK/ERK signaling which are crucial to cell proliferation, migration, and invasion. PI3K expression and Akt phosphorylation was significantly reduced in GL-V9-treated cells, yet the abundance of total Akt did not show evident change (Fig. 5). By contrast, GL-V9 showed little effect on the expression of p38 MAPK and ERK1/2 in total and phosphorylation forms (Fig. 5). These findings suggest that GL-V9 probably inhibits CRC cell migration and invasion by interfering with PI3K/Akt signaling.

### Involvement of PI3K/Akt pathway in GL-V9-mediated suppression of MMPs

**Figure 6:**
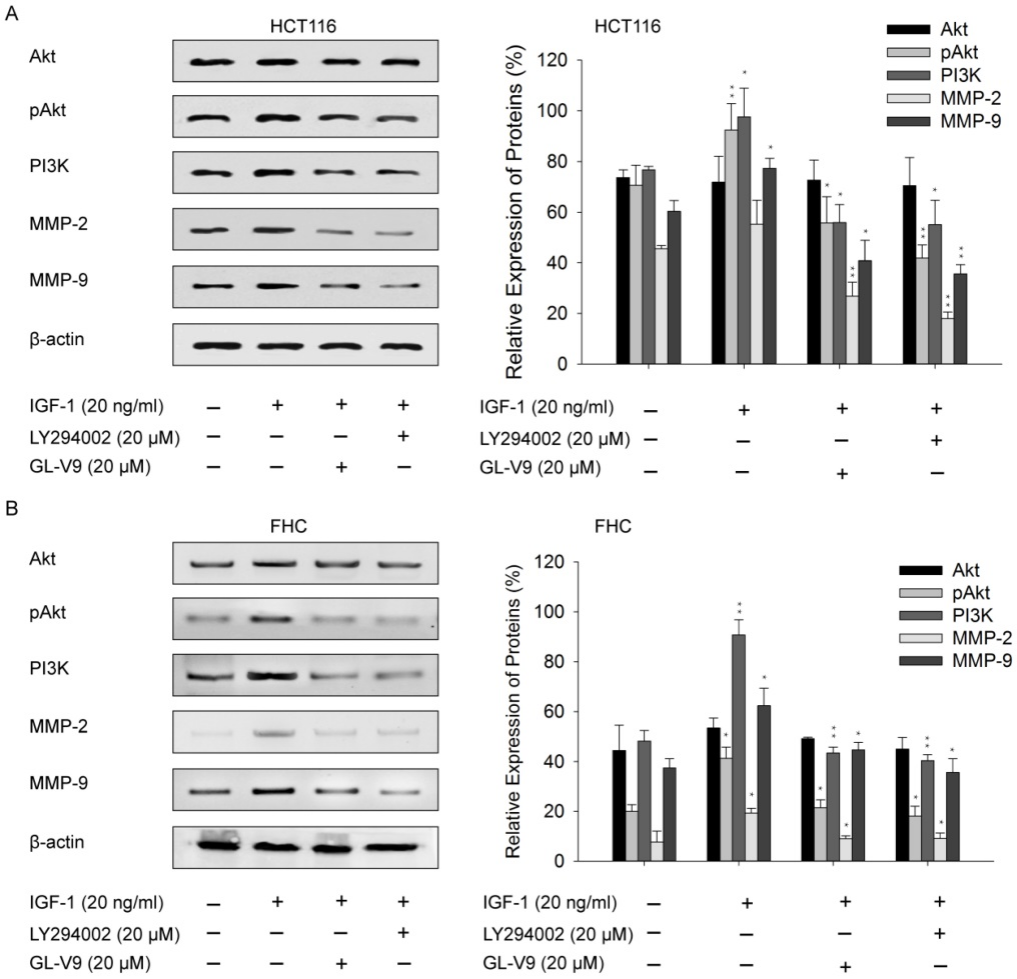
** Effect of GL-V9 on IGF-1 induced PI3K/Akt activation. (A)** CRC cells HCT116 and **(B)** normal colon epithelial cells FHC were pre-treated with 20 μM GL-V9 for 24 h and exposed to 20 ng/ml IGF-1 for 2 h. Cells pre-treated with 20 μM LY294002 for 24 h before exposure to IGF-1 were used as positive control. IGF-1 treatment led to increased PI3K expression, enhanced Akt phosphorylation, and up-regulated MMP-2 and MMP-9 expression, while pre-treatment of either GL-V9 or LY294002 significantly reversed IGF-1 induced activation of PI3K/Akt signaling and up-regulation of MMP2 and MMP-9. Relative intensities of western blot bands from three replicated experiments were quantified using the ImageJ software. The expression levels were normalized by β-actin expression. Statistical significances between the IGF-1 treated group and control group, the IGF-1 treated group and IGF-1 + LY294002/GL-V9 treated groups were calculated. Each error bar represents the mean ± S.D. of three replicate samples. * *p* < 0.05, ** *p* < 0.01.

Given the regulatory effect of GL-V9 on PI3K/Akt signaling, we sought to examine whether the PI3K/Akt signaling pathway was involved in GL-V9-induced down-regulation of MMP-2 and MMP-9. CRC cells HCT116 and normal colon cells FHC were exposed to IGF-1, a PI3K/Akt signaling activator. After treatment of 20 ng/ml of IGF-1 for 2 h, the expression of pAkt, PI3K, MMP-2, and MMP-9 were significantly increased (Fig. 6). However, this effect was reversed when the cells were pre-treated with 20 μM GL-V9 for 24 h followed by IGF-1 treatment (Fig. 6). On the contrary, a specific PI3K inhibitor, LY294002, significantly inhibited Akt phosphorylation and reduced PI3K, MMP-2, and MMP-9 expression. Cells pre-treated with 20 μM LY294002 for 24 h before exposure to IGF-1 for 2 h showed similar changes in the activity of PI3K/Akt signaling as well as MMP-2/-9 expression with those pre-treated with GL-V9 (Fig. 6). As expected, the expression levels of Akt, pAkt, PI3K, MMP-2, and MMP-9 were lower in normal cells than in CRC cells, yet either IGF-1 treatment or combined treatment of IGF-1+LY294002/GL-V9 led to similar but less evident changes in the expression of these key proteins (Fig. 6). Taken together, the inhibitory effect of GL-V9 on MMP-2 and MMP-9 may result from suppression of the PI3K/Akt signaling pathway.

## Discussion

Wogonin (5,7-dihydroxy-8-methoxyflavone) is a mono-flavonoid isolated from the traditional Chinese medicine *Scutellaria radix* with various therapeutic potential including anti-inflammatory, anti-oxidative, anticancer activities 21-24. The high selectivity of wogonin between normal cells and cancer cells makes it a promising agent for cancer treatment 25-27. As a flavonoid derivative, GL-V9 was synthesized from wogonin through two steps as previous described 16. In recent years, several studies have demonstrated the beneficial action of GL-V9 on multiple cancer types through regulation of cell apoptosis, cell growth, inflammatory response, and drug resistance 16,18,19,28,29. Apart from its anti-cancer effect, GL-V9 can also ameliorate dextran sulfate sodium (DSS)-induced colonic oxidative stress 30. Notably, GL-V9 induces the similar biological effects as wogonin at much lower concentrations. In human hepatocellular carcinoma (HCC) cells HepG2, wogonin and GL-V9 effectively induces cell apoptosis at a concentration of 80 μM and 20 μM, respectively 16. Here we showed that GL-V9 significantly inhibits the invasive and metastatic properties of CRC cells by regulating MMP-2 and MMP-9 expression via PI3K/Akt signaling. To our knowledge, this is the first study to investigate the anti-invasive effect of GL-V9 on CRC cells.

Results from MTT assay showed that GL-V9 inhibited cell viabilities of four CRC cell lines, HCT116, SW480, SW620, and LS174T (Fig. 1). After 24 h-treatment of 20 μM GL-V9, the inhibitory rates of the four CRC cell lines varied from 15.6% to 28.5%, but the inhibitory rate of normal colon epithelial cells was lower than 10% (Fig. 1). The results indicated the specificity of GL-V9 toward CRC cells. Consistently, a previous study also showed that GL-V9 has little cytotoxic effect on normal bronchial epithelium cells even at a concentration of 80 μM, suggesting the potential of CL-V9 to target cancer cells without affecting normal tissue 28.

The transwell assay, cell wound healing assay, and cell attachment assay were used to evaluate the effect of GL-V9 on the metastatic potential of CRC and normal colon epithelial cells. The results showed that GL-V9 treatment significantly reduced CRC cell invasion, migration, and adhesion (Fig. 2, 3). Since the malignant degree of normal colon cells are much lower than CRC cells, GL-V9 showed less evident, but still significant inhibitory effect on the migratory, invasion, and adhesive potential of normal cells (Fig. 2, 3). In agreement with our results, the efficacy of GL-V9 as well as its parent compound wogonin in inhibiting the invasive and migratory capacities of melanoma cells, breast cancer cells and oral cancer cells has been demonstrated in previous studies 17,31,32. *In vitro* experiments from both our study and previous studies indicated that GL-V9 inhibited cell migration significantly at a concentration ≤10 μM. By contrast, a concentration higher than 30 μM is necessary for wogonin to efficiently inhibit cell migration and invasion 17,31. These evidences implicated that GL-V9 may be a more potent candidate for prevention of tumor metastasis.

Tumor distant metastasis, which occurs in approximately 20% of patients at the time of initial diagnosis, is the primary cause of death in CRC 33. Elucidation of the molecular mechanism underlying CRC metastasis and identification of novel therapeutic targets have been the research hotspots in the past few years. It is well known that local invasion is the first requirement for distant metastasis, while a key event in tumor invasion is degradation of the surrounding ECM, which involves activation of MMP-2 and MMP-9 6,8. In CRC patients, expression of MMP-2 and MMP-9 are significantly correlated with disease stage, long-term survival, and clinical outcome 34-37.

The MAPK/ERK and PI3K/Akt signaling pathways are pivotal for many fundamental cellular processes such as cell proliferation, invasion, and differentiation 38,39. It is documented that induction of MAPK/ERK or PI3K/Akt contributes to cancer cell invasion by regulating MMP-2 and/or MMP-9 40-42. In this study, we found that GL-V9 treatment reduced the expression and activities of MMP-2 and MMP-9 in CRC cells (Fig. 4). Meanwhile, expression of PI3K and the phosphorylated form of Akt was significantly decreased (Fig. 5). On the contrary, GL-V9 did not affect the expression of MAPK p38 and both the total and phosphorylated forms of ERK1/2 (Fig. 5), indicating that GL-V9 probably inhibits CRC cell invasion and MMPs expression mainly through PI3K/Akt signaling. Since involvement of the PI3K/Akt/MMP-2/9 axis in CRC metastasis has been reported before 43,44, we further investigate the effects of combined treatment using GL-V9 and Akt activator IGF-1 on the expression of MMP-2 and MMP-9. Our results confirmed that for both CRC cells and normal colon epithelial cells, activation of Akt signaling led to increased MMP-2 and MMP-9 expression, while treatment of GL-V9 or the specific Akt inhibitor LY294002 counterbalanced the effect caused by IGF-1 (Fig. 6). However, given that some other modulators of the MMPs activity, such as the hedgehog signaling and NF-κB signaling pathways have also been identified 44,45, whether the inhibitory effects of GL-V9 on MMP-2 and MMP-9 expression are solely dependent on PI3K/Akt signaling remain to be further confirmed.

In conclusion, our study demonstrated that GL-V9 could inhibit the invasion and metastasis of CRC cells *in vitro*. The inner molecular mechanisms involved inhibition of GL-V9 on the expression and activities of MMP-2 and MMP-9 possibly via suppression of the PI3K/Akt signaling. Taken together with previous studies showing its anti-tumor effect, GL-V9 appears to be a promising therapeutic agent of CRC.
